# No-U-turn sampling for fast Bayesian inference in ADMB and TMB: Introducing the adnuts and tmbstan R packages

**DOI:** 10.1371/journal.pone.0197954

**Published:** 2018-05-24

**Authors:** Cole C. Monnahan, Kasper Kristensen

**Affiliations:** 1 School of Aquatic and Fishery Sciences, University of Washington, Seattle, Washington, United States of America; 2 Departamento de Oceanografía, Universidad de Concepción, Concepción, Chile; 3 National Institute of Aquatic Resources, Technical University of Denmark, Kemitorvet, Denmark; Southwest University, CHINA

## Abstract

Statistical inference is a widely-used, powerful tool for learning about natural processes in diverse fields. The statistical software platforms AD Model Builder (ADMB) and Template Model Builder (TMB) are particularly popular in the ecological literature, where they are typically used to perform frequentist inference of complex models. However, both lack capabilities for flexible and efficient Markov chain Monte Carlo (MCMC) integration. Recently, the no-U-turn sampler (NUTS) MCMC algorithm has gained popularity for Bayesian inference through the software Stan because it is efficient for high dimensional, complex hierarchical models. Here, we introduce the R packages adnuts and tmbstan, which provide NUTS sampling in parallel and interactive diagnostics with ShinyStan. The ADMB source code was modified to provide NUTS, while TMB models are linked directly into Stan. We describe the packages, provide case studies demonstrating their use, and contrast performance against Stan. For TMB models, we show how to test the accuracy of the Laplace approximation using NUTS. For complex models, the performance of ADMB and TMB was typically within +/- 50% the speed of Stan. In one TMB case study we found inaccuracies in the Laplace approximation, potentially leading to biased inference. adnuts provides a new method for estimating hierarchical ADMB models which previously were infeasible. TMB users can fit the same model in both frequentist and Bayesian paradigms, including using NUTS to test the validity of the Laplace approximation of the marginal likelihood for arbitrary subsets of parameters. These software developments extend the available statistical methods of the ADMB and TMB user base with no additional effort by the user.

## Introduction

Frequentist and Bayesian statistical inference are powerful tools for investigating natural processes throughout diverse fields, including ecology [1,2]. The software package AD Model Builder (ADMB; [3]) has a long history, primarily in fisheries science, for complex non-linear fixed effects models, but an extension allowing estimation of mixed effects models, and an accompanying R package, led to broader use in the ecological community [4, 5]. However, manual specification of separable functions hindered the popularity of this software for complex mixed effects models. Recently, Template Model Builder (TMB; [6, 7]) was developed specifically to efficiently estimate frequentist mixed effects models using the Laplace approximation to the marginal likelihood [4], effectively replacing ADMB for such models. However, these software platforms designed for frequentist inference lack flexible, efficient (i.e., fast) capabilities for working in the Bayesian framework.

Recently, the software package Stan [8, 9] has gained popularity due to its applicability to a broad range of Bayesian models and efficient Markov chain Monte Carlo (MCMC) sampling [10]. For instance, Stan is faster than the popular JAGS software [11] for complex hierarchical ecological models [12]. Stan achieves such efficiency with the no-U-turn sampler (NUTS; [13], a self-tuning variant of Hamiltonian Monte Carlo (HMC [14, 15], a family of MCMC algorithms. Thus, ADMB and TMB models rewritten in Stan could use NUTS to gain state-of-the-art MCMC methods.

However, it is not always feasible nor desirable to rewrite a model in Stan. For instance, some ADMB models can be tens of thousands of lines of code (e.g., [16, 17]), or use features unavailable in Stan such as phased optimization. Likewise, TMB users may not want to abandon the marginal Laplace approximation as an option for frequentist inference in hierarchical models, either for philosophical or practical reasons. For instance, maximum marginal likelihood estimation with the Laplace approximation tends to be orders of magnitude faster, and thus useful during model development or for high-dimensional, challenging models. Despite this reluctance, ADMB and TMB users would benefit from fast MCMC sampling for a broad range of hierarchical models. An alternative approach is to add Stan-like capabilities (such as the NUTS algorithm and diagnostic tools) to ADMB and TMB models.

Here, we introduce new software for running NUTS for ADMB and TMB models. The R package adnuts [18] provides NUTS sampling for ADMB models, while the package tmbstan [19] does the same for TMB. We detail their capabilities, demonstrate the methods on ecological examples, and contrast their performance against Stan. Adding state-of-the-art MCMC sampling to ADMB and TMB models allows users of these platforms an expanded toolset to better analyze data and gain deeper insights into natural processes.

## Software implementation

For HMC to be efficient, it needs fast and accurate gradient calculations for arbitrary log-posteriors. Stan accomplishes this with automatic differentiation [20]. Fortunately, both ADMB and TMB also have this capability for models built in their respective “template” languages. HMC also needs optimal trajectory lengths and step sizes, and information about the posterior shape via a ‘mass matrix’ (see section 4.1 of [14]). NUTS automatically produces nearly-optimal trajectory lengths [21], and tunes the optimal step size during warmup, while Stan introduced mass matrix adaptation during warmup (see section 34.2 of [8]).

ADMB added HMC a decade ago, but it is rarely used due to tuning difficulties. However, it was a convenient starting place to build the basic NUTS framework (i.e., algorithm 6 in [13]). We then added diagonal mass matrix adaptation modeled after Stan’s. As the NUTS code is in the ADMB source, the capability is in the model executable, and can be called directly from the command line (although we discourage it). In contrast, we were able to link TMB directly to Stan using the class 'op_matrix_vari' of the rstan package [22]. Thus, the model objective (fn) and gradient (gr) functions are calculated by TMB but passed to Stan which executes the NUTS algorithm.

Accompanying Stan is a suite of tools for diagnosing convergence of NUTS chains and performing inference. For instance, the rstan R package [22] contains functions to estimate effective sample sizes (ESS) and potential scale reduction factor $\hat{R}$ [23], and plotting functions for examining chain behavior. ShinyStan is an interactive tool for visual and numerical summaries of model parameters, and is particularly useful for examining NUTS chains [24]. Since our goal was to provide broad, Stan-like functionality to ADMB and TMB users without converting models to a new language, we developed R packages to facilitate running NUTS and mirror Stan as closely as possible.

### Summary of the adnuts R package

adnuts streamlines the workflow for ADMB users compared to command line execution, including parallel execution and post-processing in R. The sample_admb function can run both NUTS and Metropolis-Hastings algorithms, and can optionally evaluate the model in the ‘mceval’ phase on post-warmup samples from merged chains. The returned fits work with Stan diagnostic tools, including ShinyStan (Table 1). In addition to adaptive diagonal mass matrix, the user can specify arbitrary dense mass matrices (or the estimated maximum likelihood covariance), which is a capability not currently available to Stan users. Thus, to use NUTS an ADMB user only needs to define a valid model (including adding priors and a proper posterior) and compilation by the newest ADMB version.

**Table 1:** Summary of key functions from R packages.

Function	Purpose
sample_admb	Run NUTS or RWM chains. Options include parallel chains, specification of the mass matrix, and fine tuning of the NUTS algorithm parameters. Returns a list containing samples (samples), NUTS meta data for each iteration (sampler_params), and other information.
extract_samples	Extract samples from fitted object, including flags whether to include warmup samples and the log posterior column.
launch_shinyadmb	Launch the interactive diagnostic tool ShinyStan for an ADMB fit for both NUTS and RWM chains.
extract_sampler_params	Extract NUTS trajectory metadata, such as acceptance probabilities, divergences, and tree depths.
pairs_admb	A modified pairs plot that works specifically for ADMB fits. Includes option to add a covariance matrix estimated by inverting the Hessian matrix evaluated at the maximum likelihood point, if it exists.
tmbstan	Wrapper to pass TMB model to function stan from the rstan package. The ‘laplace’ argument toggles the use of the Laplace approximation. Other arguments are passed on to stan.

NUTS is the no-U-turn sampler and RWM is the random walk Metropolis algorithm (the original ADMB algorithm). All functions are from the adnuts package, except tmbstan which is from the tmbstan package.

### Summary of the tmbstan R package

tmbstan facilitates linkage with the function stan while adding a few top-level options. Most importantly, univariate parameter bounds can also be passed to tmbstan as vectors (including one-sided constraints) which are then applied internally, with Jacobian adjustments, by Stan. Because tmbstan uses the stan function to sample, it returns an object of class ‘stanfit’ just like a Stan model and thus works with Stan tools automatically. Thus, getting a TMB model working with Stan only requires adding explicit priors.

TMB uses the Laplace approximation to integrate random effects, but this is usually unnecessary in a Bayesian analysis because MCMC integrates all parameters. Therefore, by default TMB will ignore the declaration of integrated parameters, but this can be changed with the tmbstan argument ‘laplace’. When enabled, TMB integrates random effects while Stan integrates fixed effects with NUTS. The posterior distribution of the fixed effects will be the same whether the Laplace approximation is enabled or not, so long as the approximation is accurate. This approach can test the accuracy of the Laplace approximation, and we demonstrate it in our case studies.

## Case studies

### ADMB model: Swallows

We demonstrate NUTS sampling in ADMB with the *swallows* model fitted to mark-recapture data from Grüebler and Naef-Daenzer [25] and further analyzed in section 14.5 of Korner-Nievergelt, Roth [26]. This model estimates state-space survival and detection with environmental covariates and three random effect components for a total of 5 fixed effects and 172 random effects.

ADMB does not support one-sided constraints natively, so we included the variance parameters in log space, and used the exponentiated version in the log-density calculations. We included the necessary Jacobian adjustment for this transformation directly in the model code. We also used non-centered random effects which can perform better for HMC in hierarchical models [12, 27, 28]. See ‘Data Accessibility’ below for information on how to access the model files, data and reproducible R scripts used herein.

We recommend placing the model executable and any necessary inputs files into a separate folder, here called ‘admb’. We begin with the default NUTS settings: 3 chains, target acceptance rate of 0.8, 2000 total iterations, 1000 warmup iterations, and adapted step size and adapted diagonal mass matrix. We also include optional arguments to run three chains on parallel cores.

fit <- sample_admb(model = 'swallows', path = 'admb', init = inits,

seeds = seeds, parallel = TRUE, cores = 3)

This model exhibits 4 divergent transitions (i.e., where the simulated Hamiltonian or total energy goes to infinity), so we rerun with an increased target acceptance rate to reduce step sizes with the argument control = list(adapt_delta = 0.9). This is the recommended approach when models exhibit divergences (see section 34.4 of [8]). The model takes longer to run, but the divergences are eliminated. Using helper functions (Table 1) we examine the fit:

sum(extract_sampler_params(fit)$divergent__)

mon <- monitor(fit$samples)

launch_shinyadmb(fit)

Due to its complex hierarchical structure, this model was extremely difficult to fit using the traditional approach for MCMC in ADMB (results not shown). We also fit this model in Stan and TMB, and found that ADMB was approximately 88% as fast as Stan (via rstan), while TMB (with tmbstan) was 126% as fast (S1 Table). Thus, this complex hierarchical model posed no issues and ADMB was able to sample efficiently.

### TMB model: Wildflower

We demonstrate NUTS for TMB with the tmbstan package using a binomial generalized linear mixed effects model for flowering success of a perennial plant. This *wildflower* model uses a long-term data set (e.g., [29]) and was analyzed in Bolker, Gardner [30]. It includes three sets of random effects, two of which are crossed. As above, we used non-centered random effects and manually transformed the variance parameters. First, we compile and link the model as is normally done:

compile('wildflower.cpp')

dyn.load(dynlib('wildflower'))

random <- c('yearInterceptEffect_raw', 'plantInterceptEffect_raw',

'plantSlopeEffect_raw'),

obj <- MakeADFun(data = data, parameters = inits[[1]], random = random)

We use the tmbstan function to sample with Stan defaults, with initial values supplied as a list of lists.

options(mc.cores = 3)

fit <- tmbstan(obj = obj, chains = 3, init = inits)

launch_shinystan(fit)

Setting the mc.cores option tells Stan to use three cores to sample in parallel. The random parameters declaration was ignored by default and they were integrated with NUTS, the same as the fixed effects. We rerun the analysis with the Laplace approximation turned on, such that Stan integrates the fixed effects with NUTS, and TMB integrates the random effects with the Laplace approximation.

fit.la <- tmbstan(obj = obj, chains = 3, init = inits, laplace = TRUE)

**Fig 1:**
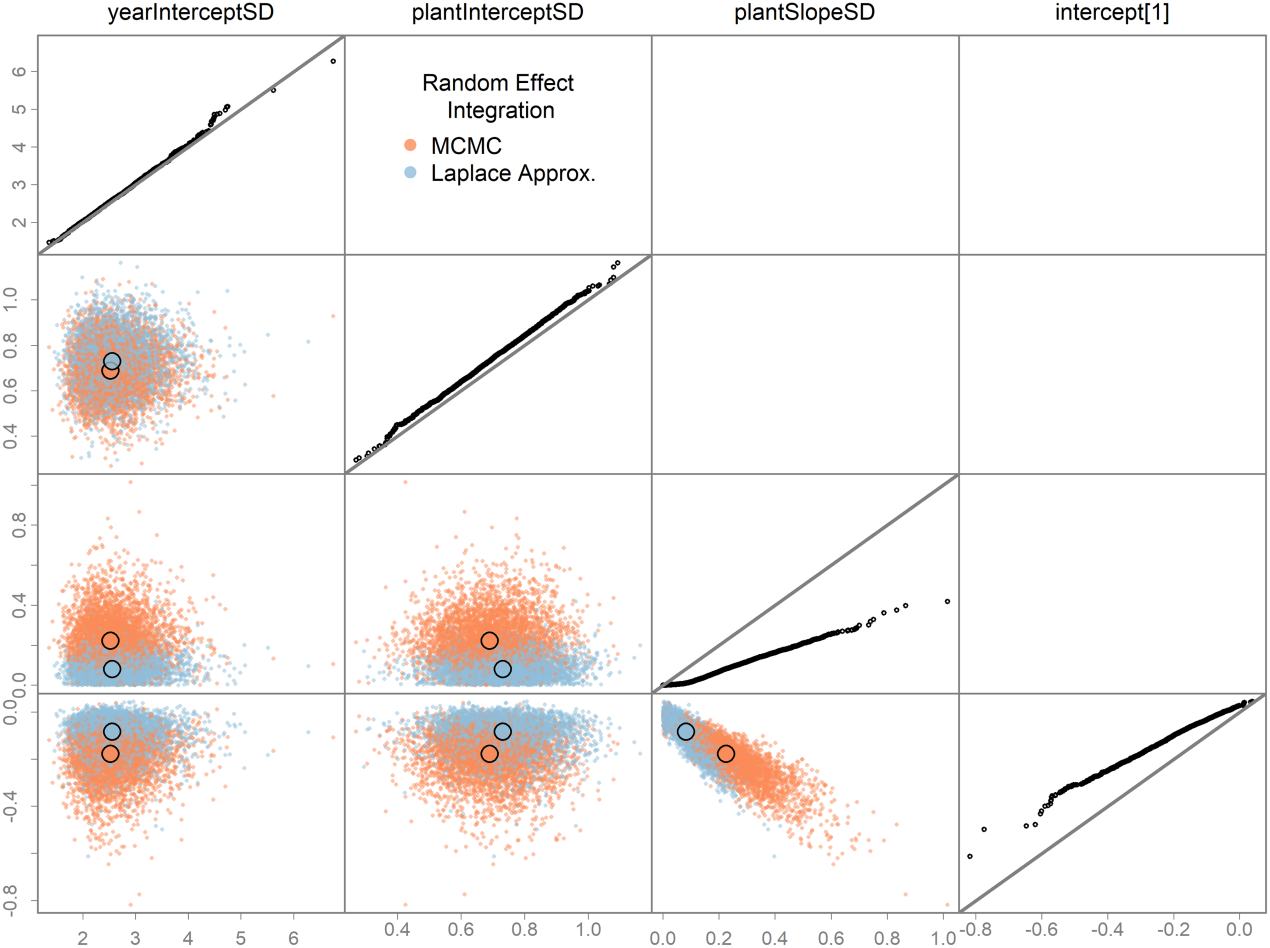
Testing the Laplace approximation of the random effects. Bayesian integration was performed on the *wildflower* TMB model with random effects integrated using two “versions”: (1) the Laplace approximation and (2) full MCMC integration via NUTS. Bayesian posterior samples of selected fixed effects (estimated with NUTS) are shown. Columns and rows corresponds to a fixed effect parameter, with the diagonal showing a QQ-plot of the two versions of the model for that parameter, including a 1:1 line in gray. Lower diagonal plots contain pairwise parameter posterior points, with color corresponding to integration version, and larger colored circles the pairwise medians. Posterior rows were randomized to prevent consistent overplotting of one version. Differences in versions suggest the Laplace approximation assumptions are not met. Other fixed effects showed no differences and are left off for clarity.

**Fig 2:**
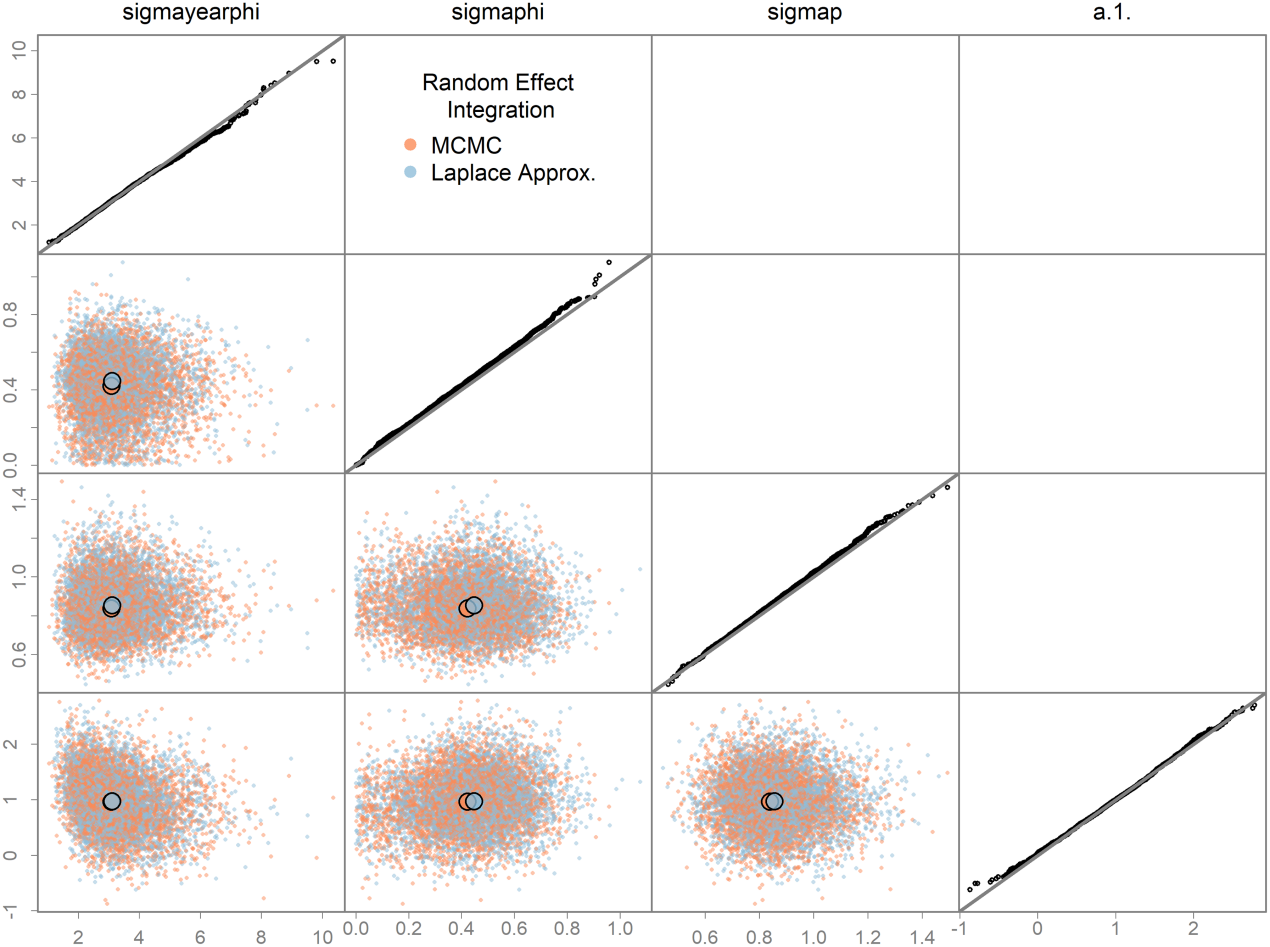
Testing the Laplace approximation integration of the random effects. Same as for Fig 1 except for three hypervariances and a slope parameter in the *swallows* model.

Here, the Laplace approximation is done at each step of each NUTS trajectory such that Stan is unaware the random effects exist. The Bayesian posteriors for two of the fixed effects differ between these two model runs (Fig 1), suggesting the Laplace approximation assumptions are not met, to a degree. This could lead to bias in parameter estimates or uncertainties. In contrast, the *swallows* model did not exhibit this property (Fig 2). Although generally not recommended with NUTS, thinning may be necessary for such tests to ensure equivalent ESS between versions, otherwise comparisons may be misleading due to different mixing rates rather than true differences. Enabling the Laplace was less efficient than full MCMC integration for the two case studies here (S2 Table), but it is unclear whether this will typically be true.

We also fit the *wildflower* model in Stan and ADMB, and found that ADMB was about 75% the speed of Stan, and TMB was 102% the speed. Thus, with only trivial changes to the TMB model template file, we obtained efficient Bayesian inference on a complex hierarchical model and tested the accuracy of the Laplace approximation.

## Discussion

Here we introduced new software which bring state-of-the-art MCMC integration to ADMB and TMB models with virtually no effort by the user. Efficiencies (i.e., effective samples per time) were relatively similar among platforms, typically within +/- 50% the speed of Stan (S1 Text). Despite this, there are some distinct advantages to doing Bayesian modeling in Stan.

We do not anticipate future developments to the ADMB NUTS code, so future algorithm advances would be unavailable. This is already true, because Stan implements exhaustive HMC [21], but this feature was not included in ADMB. Since TMB uses the Stan algorithms directly, it will not have this problem. However, Stan was developed specifically for Bayesian inference and has features in its template language that TMB users cannot use. For instance, there are more complex parameter transformations with automatic Jacobian adjustments. Thus, purely Bayesian analyses would have clear advantages by using Stan.

Whether individual models should be converted to Stan will depend. Many important ADMB models would be nearly impossible, due to extremely complex models with a suite of other software tools supporting them (e.g., [16]). For TMB, migrating to Stan means losing the ability to do the Laplace approximation on arbitrary subsets of parameters. Based on our performance tests (S1 Table, S1 Fig), substantial speed improvements by converting models to the Stan language is not guaranteed. ADMB and TMB users considering converting a model to Stan can now explore NUTS to better gauge the expected advantages.

Hierarchical modeling is clearly a powerful modeling tool for exploring ecological processes in complex data [1]. Previously, these models were difficult to estimate in ADMB and hypervariances were often fixed arbitrarily, and penalized maximum likelihood employed. Now these models can be estimated efficiently in a Bayesian framework using NUTS. TMB can already fit mixed effects models using marginal maximum likelihood via the Laplace approximation, but now users can do a full Bayesian analysis as well. In addition, the Laplace approximation can be tested by running NUTS with it turned on and off (Figs 1 and 2).

## Conclusions

These packages provide new estimation methods for thousands of existing models, primarily in ecological fields. We expect NUTS to be useful particularly within the fisheries science community, by converting penalized likelihood ADMB models to be fully Bayesian. TMB users now have access to two state-of-the-art forms of integration: the Laplace approximation and NUTS sampling as performed by Stan. We expect many analysts to adopt this framework given its flexibility in inference for a wide range of models. To our knowledge, TMB is the only software platform capable of toggling between integration tools so effortlessly. We believe that by adding powerful Bayesian integration capabilities to these two model building tools, analysts will have new tools to better investigate natural processes.

## Supporting information

S1 Text Speed comparisons between ADMB, TMB, and Stan. Further details of how we compared the efficiency (effective samples per time) for a suite of models across the three software platforms.
(DOCX)

S1 Fig Results of simulation examples with increasing dimensionality. Rows show different metrics: runtime (in seconds) includes warmup and sampling iterations but not compilation, ESS is the minimum effective sample size, and efficiency is ESS/runtime. Columns show different models: *zdiag* is independent normal but variable variances, *growth* is a non-linear mixed effects model with increasing numbers of animals. Lines denote median across 30 chains intitialized from diffuse points.
(PNG)

S2 Fig Results of empirical models. Median and interquartile range (points and vertical lines) across 30 chains.
(PNG)

S1 Table Performance on empirical models of NUTS across three platforms using default settings. Efficiencies are relative to Stan for each model, across 30 replicates with the same diffuse initial conditions. Stan uses the package rstan, TMB models used package tmbstan and ADMB models package adnuts.
(DOCX)

S2 Table Performance of versions of inference for the swallows and wildflower model. The TMB models were estimated in three ways: marginal maximum likelihood with the Laplace approximation (MLE), Bayesian integration of all parameters with NUTS using tmbstan (Full Bayesian), and Bayesian integration of fixed effects using tmbstan while using the Laplace approximation for the random effects (Laplace).
(DOCX)
