# Supplementary material for: No-U-turn sampling for fast Bayesian inference in ADMB and TMB: Introducing the adnuts and tmbstan R packages
Source: PLoS One. 2018 May 24;13(5):e0197954. doi: 10.1371/journal.pone.0197954 (PMC5967695; doi:10.1371/journal.pone.0197954)
Supplement: S1 Text — Further details of how we compared the efficiency (effective samples per time) for a suite of models across the three software platforms. (DOCX) [file pone.0197954.s001.docx]

Comparing performance between Stan, TMB and ADMB

# Comparing efficiency

Performance of Bayesian integration is the rate at which effective samples are generated. Generally this is calculated as the minimum estimated effective sample size (defined as ‘ESS’, across parameters, and as calculated by rstan::monitor), divided by the total run time (excluding compilation). Stan has been shown to outperform other software platforms like JAGS and Stata [[1](#_ENREF_1), [2](#_ENREF_2)]. It general it is difficult to accurately and fairly compare Bayesian efficiencies due to the stochastic nature of MCMC, noisy estimation of ESS, influence of posterior geometry on sampling, and the particular efficiencies of a templated language. Despite this, our goal here was to estimate rough performance differences of NUTS across Stan, TMB, and ADMB across a set of models.

For hierarchical models, we used the non-centered parameterization for all platforms [[1](#_ENREF_1), [3](#_ENREF_3), [4](#_ENREF_4)]. The different template languages inherently lead to slightly different approaches for calculating the log-density, but we ensured that the posteriors were identical across platforms for the same model. We also tried to use efficient coding practices, such as Stan’s vectorized log-likelihood calculations, where possible. All model files and R code to reproduce the simulation study can be found online (see main text).

We tested both simulated models, which are easy to increase dimensionality, and real ecological models, similar to those used in [[1](#_ENREF_1)]. We fit two simulated models with increasing dimensionality. First, were independent normal distributions with variances *1/N, 2/N, …, N/N* for a model with *N* dimensions, named *zdiag*. Off-diagonals were set to zero. Large differences in marginal variances cause inefficiencies for HMC, so these models are more difficult to fit as *N* increases beyond the increase in dimensionality. However, the diagonal adaptation of the mass matrix counteracts this, so in a way these models also test the adaptation abilities of the platforms. Our second simulated model was a non-linear generalized linear mixed effects model, fitted to simulated somatic growth trajectories (repeated measures) of individual animals. Each individual fish has two random effects controlling growth, so increasing the number of observed fish increases the number of random effects.

In addition to the empirical models *swallows* and *wildflower* described in the main text, we fit a state-space logistic fisheries population dynamics model, *logistic*. This model was originally published in [Millar and Meyer (5](#_ENREF_5)], but also a test case in [Monnahan, Thorson (1](#_ENREF_1)]. It has 28 parameters (22 random effects) and is fitted to catch per unit effort data.

For all tests we used default settings (2000 iterations, 1000 warmup, no thinning, diagonal mass matrix adaptation and step size adaptation). Where necessary we increased the target acceptance rate to eliminate divergences. We ran 30 chains initialized at the same diffuse value for each platform, and calculated the efficiency for each chain independently for each platform. Overall relative performance for each model was calculated as the median (across chains) performance of a platform divided by the median performance of Stan.

# Results

For the simulated normal *zdiag*, the ESS was similar for Stan and tmbstan and noticeably higher than ADMB for all dimensions (S1 Fig). This is likely due to the Exhaustive HMC extension implemented in Stan, and hence tmbstan [[6](#_ENREF_6)]. ESS was also relatively constant with increasing dimensionality for this model for all platforms. For the *zdiag* model Stan had the smallest runtime so it was more efficient than tmbstan. For the simulated growth model, the differences in ESS were minimal among platforms, but decreased with dimension. As before, run time was smallest with Stan and it was therefore the fastest.

The three empirical models showed slightly different patterns. For the smallest model, *logistic*, ADMB was clearly the least efficient (S2 Fig). However, efficiencies were relatively similar for the swallows and *wildflower* models, with overlap among the three platforms among chains, but tmbstan being about 20% and 2% more efficient than Stan respectively (Table S1).

# References

1. Monnahan CC, Thorson JT, Branch TA. Faster estimation of Bayesian models in ecology using Hamiltonian Monte Carlo. Methods in Ecology and Evolution. 2017;8(3):339-48. doi: 10.1111/2041-210X.12681. PubMed PMID: ISI:000396017400008.

2. Grant RL, Furr DC, Carpenter B, Gelman A. Fitting Bayesian item response models in Stata and Stan. arXiv preprint arXiv:160103443. 2016.

3. Papaspiliopoulos O, Roberts GO, Skold M. A general framework for the parametrization of hierarchical models. Stat Sci. 2007;22(1):59-73. doi: 10.1214/088342307000000014. PubMed PMID: ISI:000249036700007.

4. Betancourt M, Girolami M. Hamiltonian Monte Carlo for hierarchical models. Current Trends in Bayesian Methodology with Applications. 2015:79.

5. Millar RB, Meyer R. Non-linear state space modelling of fisheries biomass dynamics by using Metropolis-Hastings within-Gibbs sampling. J Roy Stat Soc C-App. 2000;49:327-42. doi: Doi 10.1111/1467-9876.00195. PubMed PMID: ISI:000088295400003.

6. Betancourt M. Identifying the optimal integration time in Hamiltonian Monte Carlo. arXiv preprint arXiv:160100225. 2016.
