# Supplementary figures and images for: No-U-turn sampling for fast Bayesian inference in ADMB and TMB: Introducing the adnuts and tmbstan R packages
Source: PLoS One. 2018 May 24;13(5):e0197954. doi: 10.1371/journal.pone.0197954 (PMC5967695; doi:10.1371/journal.pone.0197954)

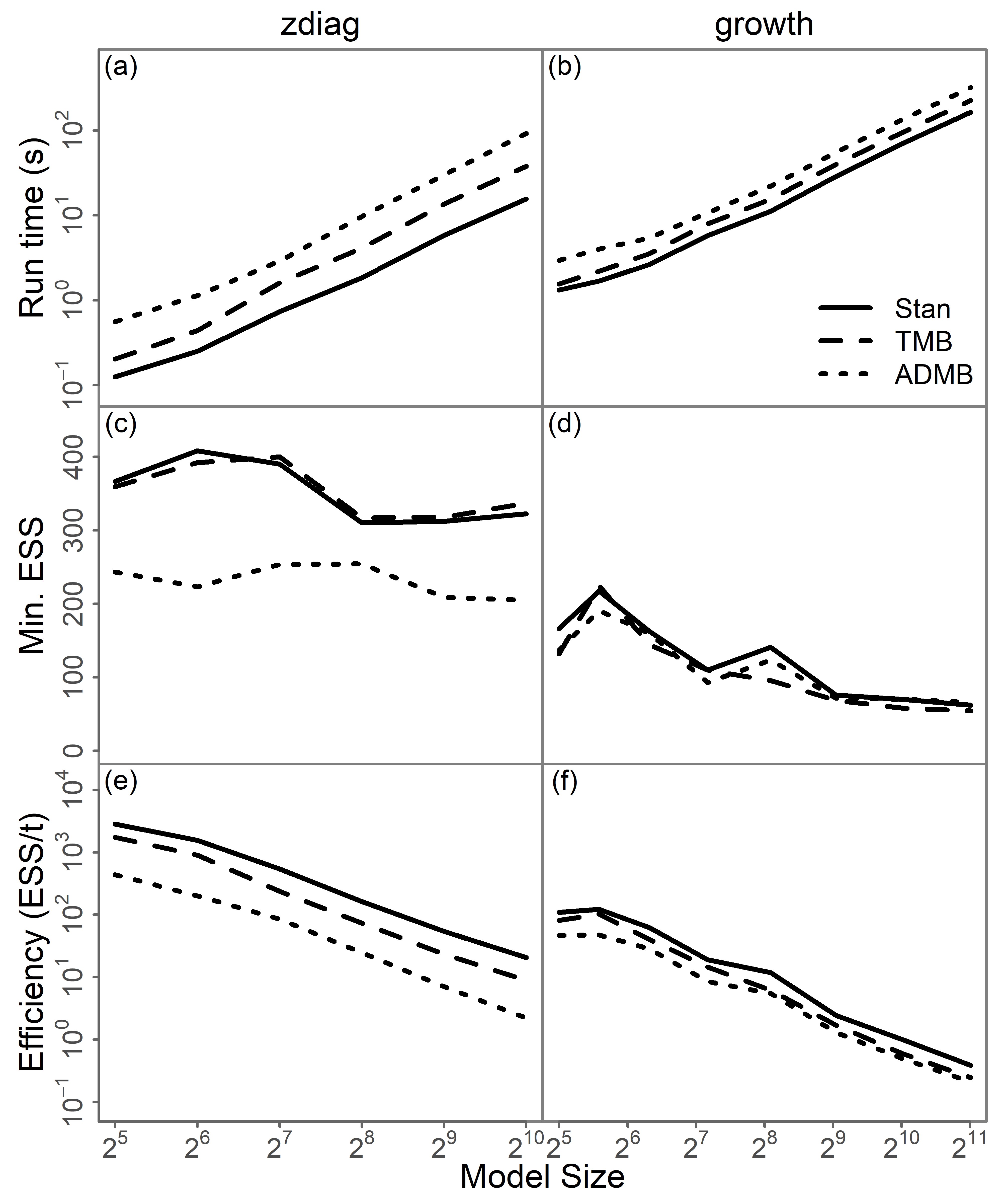

Supplement: S1 Fig — Rows show different metrics: runtime (in seconds) includes warmup and sampling iterations but not compilation, ESS is the minimum effective sample size, and efficiency is ESS/runtime. Columns show different models: zdiag is independent normal but variable variances, growth is a non-linear mixed effects model with increasing numbers of animals. Lines denote median across 30 chains intitialized from diffuse points. (PNG) [file pone.0197954.s002.png]

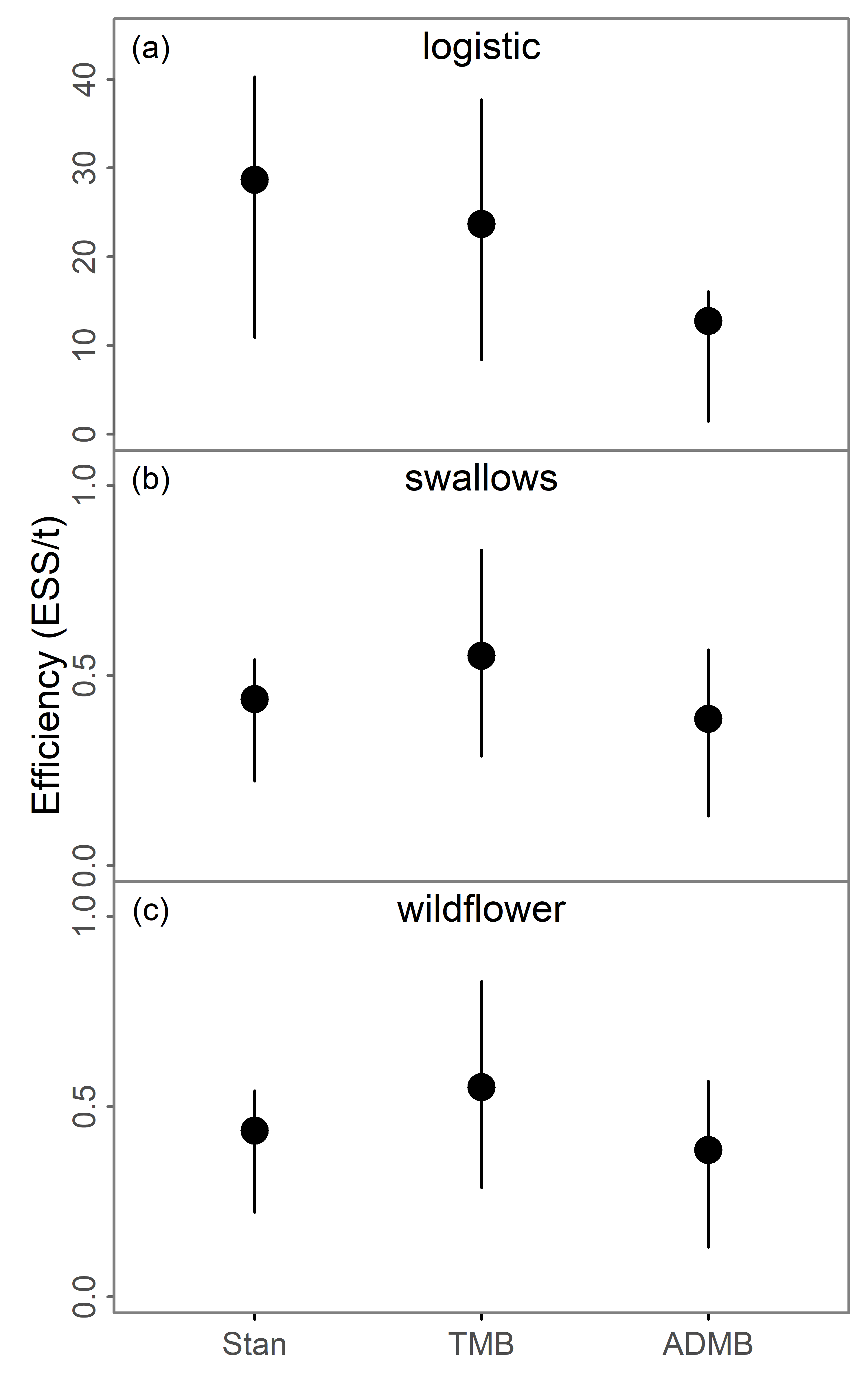

Supplement: S2 Fig — Median and interquartile range (points and vertical lines) across 30 chains. (PNG) [file pone.0197954.s003.png]
