# Supplementary material for: No-U-turn sampling for fast Bayesian inference in ADMB and TMB: Introducing the adnuts and tmbstan R packages
Source: PLoS One. 2018 May 24;13(5):e0197954. doi: 10.1371/journal.pone.0197954 (PMC5967695; doi:10.1371/journal.pone.0197954)
Supplement: S1 Table — Efficiencies are relative to Stan for each model, across 30 replicates with the same diffuse initial conditions. Stan uses the package rstan, TMB models used package tmbstan and ADMB models package adnuts. (DOCX) [file pone.0197954.s004.docx]

| **Model** | **Stan** | **TMB** | **ADMB** |
| --- | --- | --- | --- |
| Logistic | 1.00 | 0.83 | 0.45 |
| Swallows | 1.00 | 1.26 | 0.88 |
| Wildflower | 1.00 | 1.02 | 0.75 |
