# Supplementary material for: No-U-turn sampling for fast Bayesian inference in ADMB and TMB: Introducing the adnuts and tmbstan R packages
Source: PLoS One. 2018 May 24;13(5):e0197954. doi: 10.1371/journal.pone.0197954 (PMC5967695; doi:10.1371/journal.pone.0197954)
Supplement: S2 Table — The TMB models were estimated in three ways: marginal maximum likelihood with the Laplace approximation (MLE), Bayesian integration of all parameters with NUTS using tmbstan (Full Bayesian), and Bayesian integration of fixed effects using tmbstan while using the Laplace approximation for the random effects (Laplace). (DOCX) [file pone.0197954.s005.docx]

| **Model** | **Inference** | **Time (s)** | **Min. ESS** | **Efficiency (ESS/t)** |
| --- | --- | --- | --- | --- |
| Swallows | MLE | 5.3 | -- | -- |
|  | Full Bayesian | 1,633.9 | 137.2 | 0.084 |
|  | Laplace | 13,308.3 | 691.1 | 0.052 |
| Wildflower | MLE | 2.4 | -- | -- |
|  | Full Bayesian | 600.1 | 283.0 | 0.472 |
|  | Laplace | 8,870.7 | 517.7 | 0.058 |
